# Evaluating Tumor Dynamics Using Circulating Tumor DNA in the Rare Cancer Anorectal Malignant Melanoma: A Report of Two Cases

**DOI:** 10.70352/scrj.cr.25-0109

**Published:** 2025-06-17

**Authors:** Goro Takahashi, Takeshi Yamada, Kay Uehara, Seiichi Shinji, Akihisa Matsuda, Yasuyuki Yokoyama, Takuma Iwai, Toshimitsu Miyasaka, Shintaro Kanaka, Yuta Kikuchi, Takanori Matsui, Koki Hayashi, Hiroshi Yoshida

**Affiliations:** Department of Gastroenterological Surgery, Nippon Medical School, Bunkyo-ku, Tokyo, Japan

**Keywords:** anorectal malignant melanoma, rare cancer, circulating tumor DNA, biomarker

## Abstract

**INTRODUCTION:**

Anorectal malignant melanoma (ARMM) is an extremely rare and aggressive cancer that lacks specific tumor markers, making tumor dynamics difficult to monitor. Circulating tumor DNA (ctDNA), which contains cancer-specific gene mutations, has emerged as a promising biomarker for monitoring various malignant tumors. We herein report the clinical utility of ctDNA measurements in 2 patients with ARMM.

**CASE PRESENTATION:**

Case 1: A 64-year-old woman diagnosed with clinical Stage III ARMM underwent laparoscopic abdominoperineal resection with lateral lymph node dissection. Pathological findings confirmed R0 resection, and genetic analysis identified a neuroblastoma RAS viral (v-ras) oncogene homologue (*NRAS*) mutation (p.G12S). She remained recurrence-free for 7 years postoperatively. In a longitudinal ctDNA study, the preoperative ctDNA level (28.7 copies/mL) became undetectable after curative resection and remained undetectable throughout follow-up. By contrast, lactate dehydrogenase (LDH) currently the only blood-based tumor marker for mucosal melanoma, including ARMM, remained within the normal range (124–222 U/L) both preoperatively and postoperatively. Case 2: A 69-year-old woman diagnosed with clinical Stage III ARMM underwent laparoscopic abdominoperineal resection with lateral lymph node dissection. Pathological findings confirmed R0 resection, and genetic analysis identified two *KIT* proto-oncogene receptor tyrosine kinase (*KIT*) mutations (p.Y553H and p.Y646C) in exons 11 and 13. One year postoperatively, lung metastases were confirmed via positron emission tomography-computed tomography, and nivolumab monotherapy was initiated. The lung metastases remained stable for 7 months. In a longitudinal ctDNA study, the preoperative ctDNA level (28.7 copies/mL) became undetectable following curative resection. However, ctDNA re-emerged (9.05 and 7.2 copies/mL, respectively) at the time of lung metastasis detection. After initiating nivolumab treatment, ctDNA again became undetectable. Throughout the clinical course, the LDH level remained consistently within the normal range.

**CONCLUSIONS:**

We demonstrated the utility of ctDNA monitoring in patients with ARMM, a rare tumor. This method has the potential to be applied to other rare tumors if tumor-specific mutations can be identified.

## Abbreviations

ARMM
: anorectal malignant melanoma
ctDNA
: circulating tumor DNA
dPCR
: digital polymerase chain reaction
FDG
: fluorine-18-deoxyglucose
*KIT*
: KIT proto-oncogene receptor tyrosine kinase
LDH
: lactate dehydrogenase
*NRAS*
: neuroblastoma RAS viral (v-ras) oncogene homologue
PET-CT
: positron emission tomography-computed tomography

## INTRODUCTION

ARMM is an extremely rare and aggressive cancer that lacks a specific tumor marker, making it challenging to assess tumor dynamics through blood tests. Currently, serum LDH is the only blood-based tumor marker used for mucosal melanoma, including ARMM. Elevated LDH levels are associated with decreased disease-specific survival.^1,2)^ However, LDH is nonspecific and can increase in the presence of various conditions, such as inflammation, trauma, and other malignancies. ctDNA, which carries cancer-specific gene mutations, has emerged as a promising biomarker for monitoring various malignancies.^3–5)^ We herein report 2 cases of ARMM in which serial ctDNA measurements provided valuable insights into tumor dynamics.

## CASE PRESENTATION

### Case 1

**Fig. 1:**
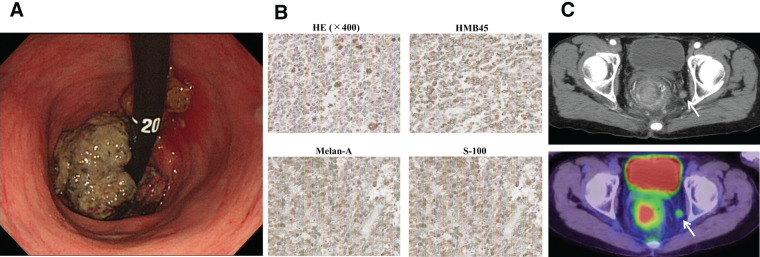
Diagnostic findings in Case 1. (**A**) Colonoscopy revealed a black ulcerated tumor extending from the anal canal to the lower rectum. (**B**) HE staining showed tumor cells with pleomorphic nuclei and melanin granules. Immunohistochemical staining confirmed malignant melanoma, with tumor cells positive for HMB-45, Melan-A, and S-100. (**C**) Positron emission tomography-computed tomography imaging demonstrated fluorine-18-deoxyglucose uptake in the primary lesion (maximum standardized uptake value of 13.6) and left pelvic lymph nodes (white arrow). HE, hematoxylin–eosin; HMB-45, human melanoma black-45; Melan-A, melanoma antigen recognized by T cells 1; S-100, S100 calcium-binding protein

A 64-year-old woman underwent colonoscopy because of a 2-month history of anal pain and bleeding during defecation. The examination revealed a black, circumferential tumor with superficial necrotic tissue in the anal canal (**Fig. 1A**). Hematoxylin and eosin staining of biopsy specimens suggested ARMM, which was subsequently confirmed by immunohistochemistry using specific markers (**Fig. 1B**). Positron emission tomography-computed tomography (PET-CT) demonstrated FDG uptake in the primary tumor and the left pelvic lymph nodes (**Fig. 1C**). Based on the Japanese Society for Cancer of the Colon and Rectum 9th edition cancer staging system,^6)^ the clinical diagnosis was Stage IIIc (cT3N3M0) ARMM. The patient underwent laparoscopic-assisted abdominoperineal resection with pelvic lymph node dissection, and pathology confirmed R0 resection. The final diagnosis was fStage IIIc (fT2N3M0) ARMM. Remarkably, she remained recurrence-free for 7 years following surgery without adjuvant chemotherapy.

**Fig. 2:**
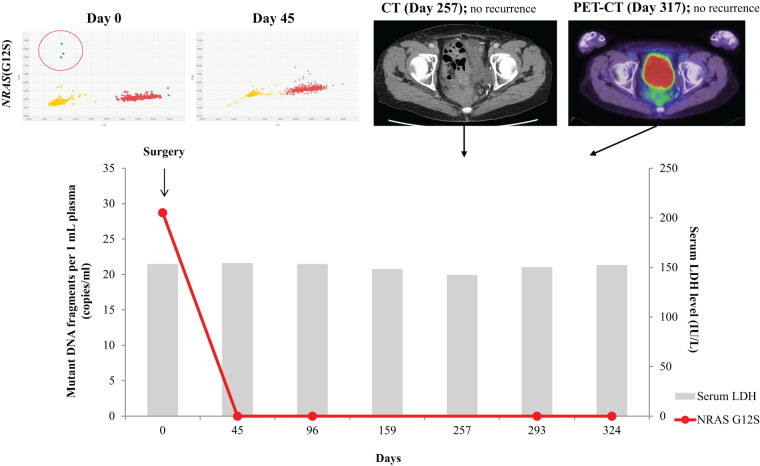
ctDNA and LDH levels during clinical course in Case 1. In a longitudinal ctDNA study, the preoperative ctDNA level was 28.7 copies/mL, which became undetectable after curative resection and remained undetectable throughout follow-up. Furthermore, there were no findings of recurrence on the CT or PET-CT, confirming the absence of recurrence. By contrast, the LDH levels remained stable throughout the clinical course. dPCR results: blue dots represent ctDNA (*NRAS* G12S), while red dots indicate wild-type fragments. CT, computed tomography; ctDNA, circulating tumor DNA; dPCR, digital polymerase chain reaction; LDH, lactate dehydrogenase; *NRAS*, neuroblastoma RAS viral (v-ras) oncogene homologue; PET, positron emission tomography

In a longitudinal study analyzing ctDNA, we utilized ctDNA as a monitoring biomarker in Case 1 (**Fig. 2**). Next-generation DNA sequencing (NGS) of the primary tumor, performed using the Ion AmpliSeq Cancer Hotspot Panel v2 (Thermo Fisher Scientific, San Francisco, CA, USA), identified a neuroblastoma RAS viral (v-ras) oncogene homologue (*NRAS*) mutation (p.G12S). This tumor-specific mutation was determined by comparing DNA-sequencing results from the primary tumor tissue and corresponding leukocytes, and it was subsequently used as a monitoring biomarker. Circulating cell-free DNA was extracted from 1 mL of plasma, and ctDNA within the circulating cell-free DNA was detected using a dPCR system. The ctDNA level, which was 28.7 copies/mL before surgery, became undetectable following curative resection and remained undetectable throughout follow-up. By contrast, LDH levels remained within the normal range (124–222 U/L) both preoperatively and during follow-up.

### Case 2

**Fig. 3:**
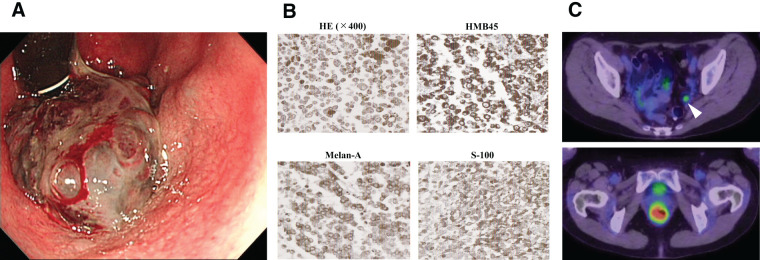
Diagnostic findings in Case 2. (**A**) Colonoscopy revealed a black elevated tumor with oozing blood in the anorectal region. (**B**) HE staining identified pleomorphic tumor cells containing melanin granules. Immunohistochemical staining confirmed malignant melanoma, with positivity for HMB-45, Melan-A, and S-100. (**C**) Positron emission tomography-computed tomography imaging demonstrated fluorine-18-deoxyglucose uptake in the primary lesion (maximum standardized uptake value of 7.88) and left pelvic lymph nodes (arrowhead). HE, hematoxylin–eosin; HMB-45, human melanoma black-45; Melan-A, melanoma antigen recognized by T cells 1; S-100, S100 calcium-binding protein

A 69-year-old woman presented with persistent diarrhea and frequent defecation for 6 months despite medication. Colonoscopy revealed a black tumor measuring 30 mm in diameter in the anorectal area, with 50% circumferential involvement (**Fig. 3A**). Immunohistochemistry of biopsy specimens confirmed malignant melanoma (**Fig. 3B**). PET-CT demonstrated FDG accumulation in both the primary tumor and the left pelvic lymph nodes (**Fig. 3C**). Following a clinical diagnosis of Stage IIIc (cT2N3M0) ARMM, the patient underwent laparoscopic-assisted abdominoperineal resection with lateral lymph node dissection. The final diagnosis was fStage IIIc (fT3N3M0) ARMM. One year after curative resection, PET-CT confirmed multiple lung metastases, and nivolumab monotherapy was initiated. The disease remained stable for 7 months following nivolumab treatment; however, therapy was discontinued because of cerebral hemorrhage.

**Fig. 4:**
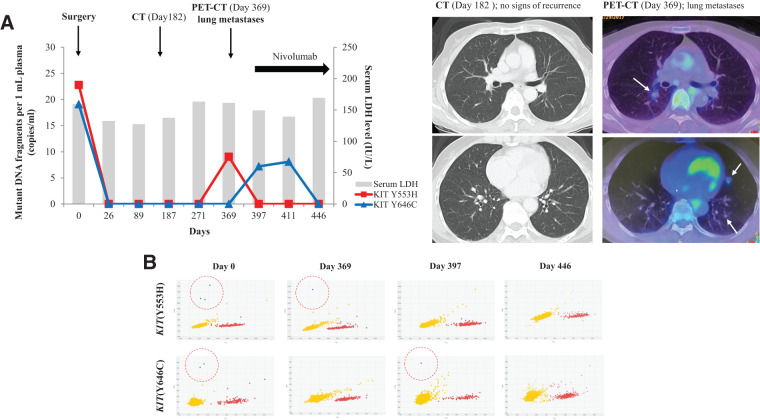
ctDNA and LDH levels during clinical course in Case 2. (**A**) In a longitudinal ctDNA study, preoperative ctDNA levels for *KIT* Y553H and *KIT* Y646C were 22.8 and 19.1 copies/mL, respectively, and became undetectable after curative resection. One year postoperatively, PET-CT revealed multiple lung metastases, and ctDNA reappeared (9.05 and 7.2 copies/mL, respectively). Following nivolumab administration, ctDNA again became undetectable (Day 446). By contrast, the LDH levels remained stable throughout the clinical course. (**B**) dPCR results: blue dots represent ctDNA (*KIT* Y553H and *KIT* Y646C), while red dots indicate wild-type fragments. CT, computed tomography; ctDNA, circulating tumor DNA; dPCR, digital polymerase chain reaction; *KIT*, KIT proto-oncogene receptor tyrosine kinase; LDH, lactate dehydrogenase; PET-CT, positron emission tomography-computed tomography

A longitudinal ctDNA analysis was performed in Case 2 using the same approach as in Case 1 (**Fig. 4**). The tumor harbored 2 *KIT* mutations (p.Y553H and p.Y646C), both of which were confirmed by Sanger sequencing (**Supplementary Fig. 1**). Prior to surgery, both ctDNA mutations were detectable at 22.8 and 19.1 copies/mL, respectively, but became undetectable after curative resection. One year postoperatively, when lung metastases were detected, the ctDNA levels for ^Y553H^*KIT* and ^Y646C^*KIT* were 9.05 and 7.2 copies/mL, respectively. Following nivolumab administration, ctDNA became undetectable on Days 397 and 446 and remained below the detection limit until treatment was discontinued because of cerebral hemorrhage. By contrast, the LDH levels remained within the normal range throughout the clinical course.

## DISCUSSION

This study has revealed 2 key clinical findings. First, ctDNA is detectable in ARMM, a rare tumor. Second, ctDNA serves as a more effective monitoring marker for ARMM than serum LDH levels.

ARMM is a rare subtype of melanoma that originates in the anorectal region, accounting for less than 1% of all melanoma cases and approximately 4% of anorectal malignancies.^7–9)^ Its symptoms, including rectal bleeding, pain, and changes in bowel habits, are nonspecific and often mistaken for other anorectal conditions, leading to delays in diagnosis. Because of its rarity, ARMM is frequently diagnosed at an advanced stage, contributing to a poor prognosis.^7–9)^ The 5-year overall survival rate for ARMM is markedly low at 20%, compared with 80% for cutaneous melanoma.^7–9)^ The standard treatment for resectable ARMM involves surgical resection, including wide local excision or endoscopic resection; however, the high risk of metastasis and recurrence remains a major challenge.^2,8–10)^ Given the aggressive nature of ARMM, identifying precise biomarkers for monitoring disease progression is crucial, particularly for rare and rapidly progressing cancers.

First, we successfully detected ctDNA in ARMM in both cases after identifying cancer-specific somatic mutations using next-generation sequencing. While mutations in v-raf murine sarcoma viral oncogene homologue B (*BRAF*), *RAS*, and neurofibromin 1 (*NF1*) are frequently observed in cutaneous malignant melanoma,^11,12)^ little is known about the genetic profile of ARMM except that mutations in the *KIT* gene are found in approximately 30% of cases.^13,14)^ To address this knowledge gap, we examined somatic mutations by analyzing and comparing primary tumor DNA with germline DNA. We identified 3 somatic mutations in *NRAS* and *KIT* from primary tumors and successfully detected ctDNA using a dPCR system. Although the usefulness of copy number (CN) alterations (e.g., *KIT*, cyclin-dependent kinase 4, and cyclin D1) in cell-free DNA, including mucosal melanoma (n = 7), has been reported, the applicability of this approach was confined to cases in which a high CN ratio was observed in the primary tumor.^15)^ Therefore, our findings indicated that if tumor-specific somatic mutations can be identified, our method may be applicable to any type of rare tumor.

Second, compared with serum LDH levels, we found ctDNA to be a more effective monitoring marker for ARMM. In both cases, ctDNA was detectable preoperatively and became undetectable after curative R0 resection. In Case 2, ctDNA reappeared when lung metastases were confirmed (Day 369) and disappeared following nivolumab administration. By contrast, serum LDH levels remained within the normal range throughout the clinical course in both cases. In cutaneous malignant melanoma, ctDNA has been reported to be more useful than serum LDH levels for monitoring tumor dynamics.^12,16)^ Similarly, our findings demonstrated that ctDNA levels more accurately reflected tumor dynamics in patients with ARMM. To our knowledge, this is the 1st report to highlight the utility of ctDNA as a monitoring biomarker for ARMM.

In ctDNA analysis, there are 2 approaches: the tumor-informed approach,^17)^ which requires genetic information from the primary tumor, and the tumor-agnostic approach, which does not require such information.^18)^ We believe that for monitoring tumor dynamics, the tumor-informed approach is sufficient because it allows for a simple interpretation of the dPCR results and requires only a one-time NGS analysis of the primary tumor before ctDNA evaluation. However, if the objective is to capture emerging mutations that may serve as therapeutic targets, the tumor-agnostic approach becomes essential because it usually requires repeated NGS assessments of ctDNA. Both strategies have their respective advantages and limitations (especially in terms of cost), and the choice of approach largely depends on the patient’s treatment status and the availability of tumor tissue information.

In recent years, evidence regarding the timing of ctDNA analysis has accumulated. In colorectal cancer, the disease-free survival of patients who were ctDNA-positive at 4 weeks postoperatively was extremely poor compared to those who were ctDNA-negative (hazard ratio 10.0, *p* < 0.0001).^17)^ Moreover, since ctDNA more accurately reflected prognosis than existing biomarkers, including pathological T stage and N stage, the detection of minimal residual disease by ctDNA analysis has emerged as a pivotal component of a ctDNA-guided approach for determining postoperative treatment strategies.^17,19)^ By using the postoperative ctDNA status to tailor adjuvant therapy decisions, clinicians can potentially improve outcomes through early intervention and avoidance of overtreatment. Therefore, especially in ARMM—a cancer known to have a poorer prognosis than colorectal cancer^8)^—the detection of minimal residual disease by ctDNA analysis at 4 weeks after curative surgery is considered indispensable in surgical cases. However, given the rarity and unique biological characteristics of ARMM, further prospective studies are required to validate these findings and to optimize ctDNA-guided treatment strategies.

Several reports have demonstrated the utility of ctDNA in other rare tumors that lack protein biomarkers. In brain tumors, ctDNA was found to be more detectable in higher-grade tumors, which are more malignant, than in lower-grade tumors.^20)^ Additionally, in gastrointestinal stromal tumors, ctDNA has proven useful not only for tracking tumor dynamics but also for detecting the emergence of mutations associated with drug resistance.^21–23)^ Monitoring rare tumors without specific biomarkers typically requires frequent computed tomography or magnetic resonance imaging scans. Given that blood-based tests are noninvasive and easily repeatable, ctDNA analysis may help reduce the need for frequent radiological examinations. Further research is needed to determine whether ctDNA monitoring can not only improve our understanding of tumor dynamics but also minimize unnecessary imaging tests in rare tumors.

Recent advancements in the treatment of mucosal melanoma, including ARMM, have shown promising outcomes, particularly with the application of immunotherapy.^11)^ Immune checkpoint inhibitors, such as programmed death-1 and cytotoxic T-lymphocyte-associated protein 4 inhibitors, have demonstrated significant efficacy by leveraging the immune system’s ability to target tumor cells.^24–26)^ Additionally, adoptive cell transfer and anti-angiogenic therapy have emerged as potential therapeutic strategies for mucosal melanoma.^27)^ Adoptive cell transfer involves the *ex vivo* modification of T cells to enhance their antitumor activity, while anti-angiogenic therapy aims to inhibit tumor progression by disrupting angiogenesis.^28)^ The integration of these therapies may provide a comprehensive approach to overcoming treatment resistance.^29)^ These advancements highlight the evolving therapeutic landscape for mucosal melanoma and offer a foundation for improving patient outcomes in these aggressive malignancies.

## CONCLUSIONS

We have demonstrated the utility of ctDNA monitoring in patients with ARMM, a rare tumor. This approach may also be applicable to other rare tumors that lack specific protein biomarkers.

## SUPPLEMENTARY MATERIALS

Supplementary Fig. 1 Confirmation of 2 different *KIT* mutations using Sanger sequencing in Case 2. Two distinct *KIT* mutations (exons 11 and 13) identified by next-generation sequencing were validated through Sanger sequencing.
*KIT*, *KIT* proto-oncogene receptor tyrosine kinase

## ACKNOWLEDGMENTS

We thank Mrs. Miyako Yoshimura for providing technical support in the experiments. We also thank Angela Morben, DVM, ELS, from Edanz (https://jp.edanz.com/ac), for editing a draft of this manuscript. The funding source played no role in this study.

## DECLARATIONS

### Funding

This work was supported by the Department of Gastroenterological Surgery, Nippon Medical School, Tokyo, Japan.

### Authors’ contributions

GT drafted the main manuscript.

TY, KU, SS, AM, YK, and HY reviewed and approved the manuscript.

GT, YY, TI, T Miyasaka, and SK processed patient samples for this study.

T Miyasaka, SK, T Matsui, and KH contributed to the establishment of the dPCR system.

All authors have reviewed and approved the final manuscript.

### Availability of data and materials

The datasets supporting the conclusions of this article are included within the article.

### Ethics approval and consent to participate

Ethics approval for publication of this case report was obtained from the Ethics Committee of Nippon Medical School Hospital (Tokyo, Japan, Approval No. 28-03-738). All procedures performed in this study were in accordance with the guidelines of the Declaration of Helsinki. Written informed consent was obtained from both patients.

### Consent for publication

Written informed consent for publication of this case report and the accompanying images were obtained from the patients.

### Competing interests

The authors declare that they have no competing interests.
