# Supplementary material for: Evaluating Tumor Dynamics Using Circulating Tumor DNA in the Rare Cancer Anorectal Malignant Melanoma: A Report of Two Cases
Source: Surg Case Rep. 2025 Jun 17;11(1):25-0109. doi: 10.70352/scrj.cr.25-0109 (PMC12207099; doi:10.70352/scrj.cr.25-0109)
Supplement: Supplementary Fig. 1 — Confirmation of 2 different KIT mutations using Sanger sequencing in Case 2. Two distinct KIT mutations (exons 11 and 13) identified by next-generation sequencing were validated through Sanger sequencing. KIT, KIT proto-oncogene receptor tyrosine kinase [file scr-11-01-25-0109-s001.pptx]

## Slide 1
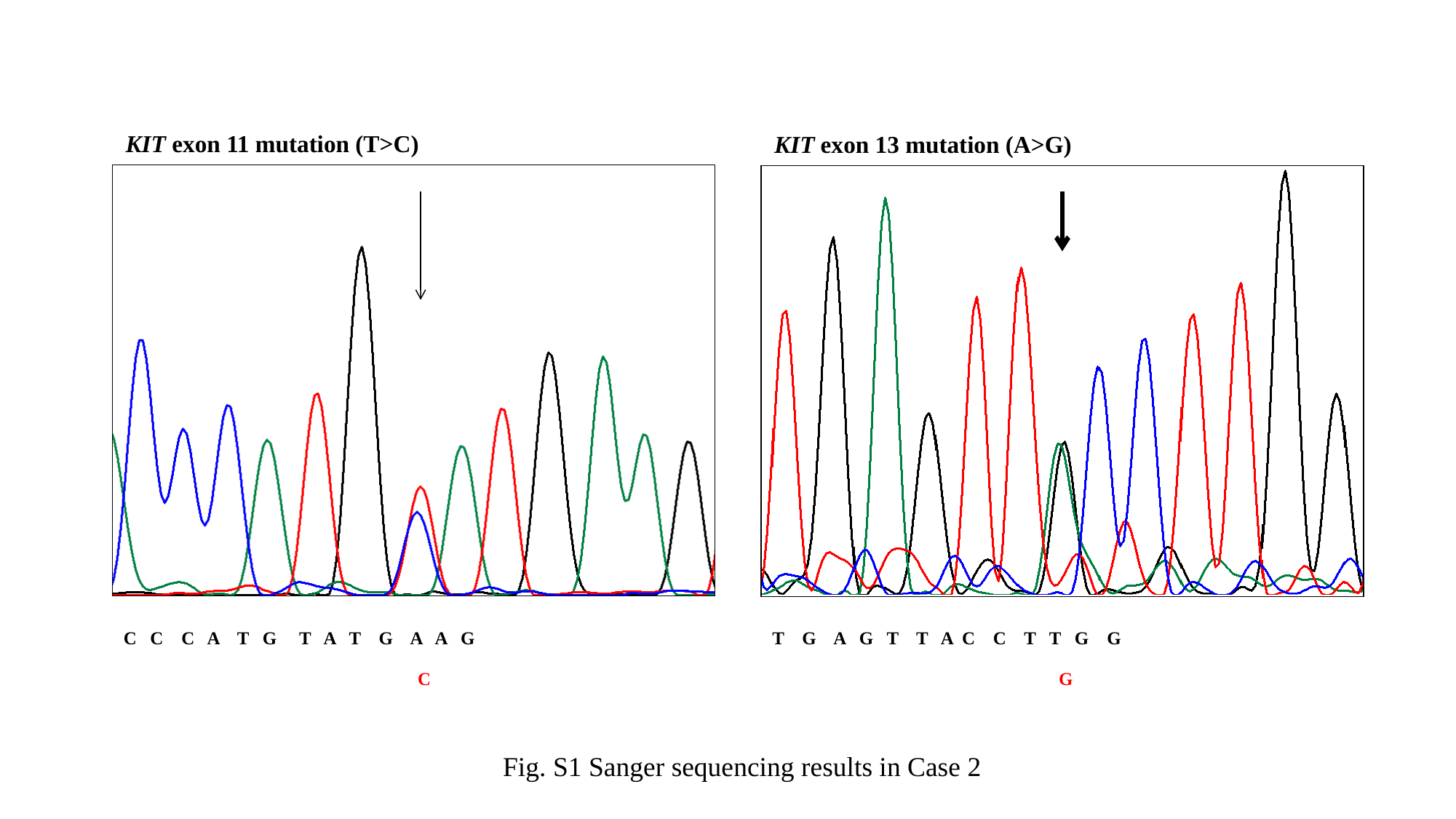

KIT exon 11 mutation (T>C)
KIT exon 13 mutation (A>G)
T G A G T T A C C T T G G
C C C A T G T A T G A A G
C
G
Fig. S1 Sanger sequencing results in Case 2
